# Upper and Lower Limb Training Evaluation System Based on Virtual Reality Technology

**DOI:** 10.3390/s24216909

**Published:** 2024-10-28

**Authors:** Jian Zhao, Hanlin Gao, Chen Yang, Zhejun Kuang, Mingliang Liu, Zhuozheng Dang, Lijuan Shi

**Affiliations:** 1 College of Computer Science and Technology, Changchun University, Changchun 130022, China; zhaojian@ccu.edu.cn (J.Z.); 221501468@mails.ccu.edu.cn (H.G.); 210701225@mails.ccu.edu.cn (C.Y.); 041940324@mails.ccu.edu.cn (M.L.); 231501504@mails.ccu.edu.cn (Z.D.); 2 Key Laboratory of Intelligent Rehabilitation and Barrier-Free for the Disabled, Changchun University, Ministry of Education, Changchun 130022, China; shilj@ccu.edu.cn; 3 Jilin Provincial Key Laboratory of Human Health Status Identification Function & Enhancement, Changchun 130022, China; 4 College of Electronic Information Engineering, Changchun University, Changchun 130012, China

**Keywords:** virtual reality, limb rehabilitation, posture recognition, skeletal coordinates, system evaluation

## Abstract

Upper and lower limb rehabilitation training is essential for restoring patients’ physical movement ability and enhancing muscle strength and coordination. However, traditional rehabilitation training methods have limitations, such as high costs, low patient participation, and lack of real-time feedback. The purpose of this study is to design and implement a rehabilitation training evaluation system based on virtual reality to improve the quality of patients’ rehabilitation training. This paper proposes an upper and lower limb rehabilitation training evaluation system based on virtual reality technology, aiming to solve the problems existing in traditional rehabilitation training. The system provides patients with an immersive and interactive rehabilitation training environment through virtual reality technology, aiming to improve patients’ participation and rehabilitation effects. This study used Kinect 2.0 and Leap Motion sensors to capture patients’ motion data and transmit them to virtual training scenes. The system designed multiple virtual scenes specifically for different upper and lower limbs, with a focus on hand function training. Through these scenes, patients can perform various movement training, and the system will provide real-time feedback based on the accuracy of the patient’s movements. The experimental results show that patients using the system show higher participation and better rehabilitation training effects. Compared with patients receiving traditional rehabilitation training, patients using the virtual reality system have significantly improved movement accuracy and training participation. The virtual reality rehabilitation training evaluation system developed in this study improves the quality of patients’ rehabilitation and provides personalized treatment information to medical personnel through data collection and analysis, promoting the systematization and personalization of rehabilitation training. This system is innovative and has broad application potential in the field of rehabilitation medicine.

## 1. Introduction

Upper and lower limb rehabilitation training is a vital part of rehabilitation medicine. It is widely used in patients with diseases such as burns, brain injuries, fractures, or trauma [1,2]. The main goal of this type of training is to restore patients’ physical exercise capabilities, enhance muscle strength and coordination due to diseases or injuries, and ultimately help patients improve their quality of daily life.

According to research and statistics in recent years, China has mainly concentrated on traffic accidents and other non-traffic accidents caused by accidents. According to a national traffic accident survey, from 2010 to 2019, more than 2,125,994 traffic accidents occurred in China. In these accidents, the proportion of patients involving upper and lower limb injury is higher. According to a national survey of Chinese fracture patients (more than 512,187 individuals), upper and lower limb injuries (such as arms, legs, and spine fractures) are more common types [3].

With the increase in patients with upper and lower limb injuries due to accidents, rehabilitation training has become an urgent demand for patients requiring rehabilitation. At present, the author’s country’s rehabilitation training mainly depends on the traditional method of rehabilitation, and it usually involves one-to-one rehabilitation guidance provided by rehabilitation trainers. Although this method can conduct rehabilitation training for the specific situations of patients under professional guidance, it has high rehabilitation training costs and patients need to frequently go to the rehabilitation center for treatment, which not only causes an economic burden but also greatly affects its convenience to patients.

With the rapid development of virtual reality (VR) technology and intelligent rehabilitation equipment, the field of rehabilitation medicine has ushered in new breakthroughs. VR technology can create a more immersive and interactive rehabilitation environment for patients. Combined with the multi-sensory experience and instant feedback mechanism, it can effectively improve the participation and motivation of patients in rehabilitation training. As a result, the rehabilitation effect is greatly improved [4]. At the same time, as a new means for rehabilitation auxiliary, rehabilitation training robots have gradually become research hotspots in the academic and clinical fields. Through accurate exercise assistance and data monitoring, they provide personalized real-time feedback in exercise recovery training, which can help the rehabilitation division for more efficient treatment and significantly improve the efficiency and effect of rehabilitation [5].

Although these rehabilitation technologies have made significant progress, their applications still have inherent limitations. First of all, patients easily lose interest and motivation in long-term rehabilitation training, resulting in insufficient input and enthusiasm for training [6]. Secondly, traditional rehabilitation training usually involves repeated actions and exercises. This monotonous training mode may make patients feel bored, reducing their participation and persistence [7]. In addition, the real-time feedback provided by traditional rehabilitation methods is relatively limited, and it is difficult for patients to understand their own rehabilitation progress and improvement points, which affects its confidence and effect [8]. At the same time, most of the traditional rehabilitation training adopts universal training schemes, which makes it difficult to make flexible adjustments according to the individual needs of patients, which may lead to poor rehabilitation effects [9]. Furthermore, traditional rehabilitation training requires a lot of time and resources, including the participation of rehabilitation professionals, equipment and venue use, which virtually increases the cost and time pressure of rehabilitation [10]. More importantly, traditional rehabilitation training is usually carried out in a controlled environment. It is difficult for patients to convert the training effect into a practical application situation in daily life, which affects the actual effect of rehabilitation progress [11]. These problems make traditional recovery methods face many challenges in practical applications, leading to a slow rehabilitation process and unstable effects. Patients’ participation and rehabilitation effects are often subject to multiple restrictions on situations, psychology, and external conditions, and it is difficult to achieve the ideal rehabilitation state.

Therefore, upper and lower limb rehabilitation training is essential for patients with exercise dysfunction caused by trauma or disease. It not only helps to restore patients’ daily activities but also significantly improves their quality of life and helps patients re-integrate into normal social life. Therefore, in the future, new technologies will be applied in upper and lower limb rehabilitation training, such as virtual reality, robotic rehabilitation equipment, and remote rehabilitation systems, which will further optimize the rehabilitation effect and promote the popularization and application of rehabilitation training.

## 2. Related Work

In recent years, the research of VR technology in rehabilitation medicine has continued to deepen, and its application field has expanded from traditional motion function to many scenarios such as neural rehabilitation, cognitive training, and remote rehabilitation. The main advantage of VR technology is that it can provide patients with a highly immersive and interactive virtual environment and stimulate patients’ initiative and training motivation through sensory feedback. The current research progress focuses on how to use VR systems to achieve personalized training and real-time monitoring of rehabilitation progress, combining them with other auxiliary technologies to optimize the rehabilitation effect and improve the efficiency of rehabilitation. In the field of sports function rehabilitation, Laver et al. [12] found that VR technology has a significant advantage in the function of improving the upper and lower limbs of stroke patients, especially in providing personalized rehabilitation tasks and real-time feedback mechanisms. By dynamically capturing patient movements and providing visual and tactile feedback, the VR system can effectively help patients correct motion errors and ensure the safety and effectiveness of training. In addition, many studies have further developed a rehabilitation platform for multi-sensory interaction. Use the visual, hearing, and tactile stimulation in virtual scenes to increase the patient’s sense of input of the task, thereby improving the rehabilitation effect [13,14,15].

In addition, virtual reality technology can also help rehabilitation professionals formulate personalized rehabilitation plans for patients to ensure that the rehabilitation process can be dynamically adjusted according to the specific needs of each patient and the progress of recovery. Lange et al.’s [16] research discussed how to design game-based rehabilitation tasks to meet a patient’s personalized needs and rehabilitation goals. Lloréns et al. [17] discussed balanced rehabilitation training through the Kinect bone tracking system so that patients could receive rehabilitation treatment in the family environment, thereby reducing their dependence on time and venue resources. At the same time, research by Merians et al. [7] pointed out that a rehabilitation system with enhanced virtual reality can save a lot of time and venue resources in rehabilitation while significantly improving the accessibility of rehabilitation. Sveistrup et al. [18] further explained the application advantage of virtual reality technology in sports rehabilitation, and believed that the combination of virtual training tasks with the patient’s daily life situation can improve the practical application and conversion effect of rehabilitation training.

With the continuous progress of modern rehabilitation technology, the application of virtual reality technology is not limited to patients with stroke rehabilitation, but has also gradually expanded to other nerve injuries, muscle and bone diseases, and remote rehabilitation training. Khokale et al. [19] researched the application of VR and AR technology in the application prospects of VR and AR technology in stroke rehabilitation, indicating that these technologies can provide remote rehabilitation services for patients in remote areas. Moreira et al. [20] conducted a systematic review of the application of head-wear display (HMD) virtual reality in the rehabilitation of upper limbs. It was found that it has the potential to improve the recovery of upper limb exercise and reduce pain, but it still requires large-scale research to confirm its effect. Maggio et al. [21] reviewed the application of a computer-based rehabilitation environment (Caren) system in neural rehabilitation. It was pointed out that although Caren shows potential advantages in the rehabilitation of Parkinson’s disease, traumatic brain injury (TBI), and multiple sclerosis patients, the current research samples are small and need to further verify its effectiveness in remote rehabilitation applications.

In the study of the combination of virtual reality and rehabilitation robot technology, Chheang et al. [4] proposed an upper limb rehabilitation framework combining VR and robotics technology. Analyzing the elbow joint action through wearable sensors significantly improves the participation and effect of patients in family rehabilitation. Brady et al. [22] studied this through an interview with a focus group, which discussed the view of physical therapist’s view of the rehabilitation of shoulder muscle skeletal pain support for VR support. The results showed that VR had the potential to manage pain and improve of patient participation, but there were still problems with security and operating convenience in practical applications.

In the field of upper and lower limb function rehabilitation, its broad potential has been demonstrated. Among them, Mohammadhadi Sarajchi et al. [23] studied a rehabilitation exoskeleton system designed for children aged 8 to 12. Six active joints (distributed in hip and knee joints) and an adjustable design were used to meet the growth needs of different children. Oblak et al. [24] investigated the combination of the Kinect(Microsoft Corporation, Redmond, WA, USA) device and the tactile feedback system in upper limb rehabilitation training, especially for the recovery of arm and hand function. Prrochnow et al. [25] further studied the neurological mechanism of Kinect in the virtual reality environment and verified its application effect in rehabilitation therapy in patients with stroke and nerve injuries. In addition, through in-depth investigations, we found that patients using Kinect devices combined with rehabilitation games for training showed greater improvements in the recovery and rehabilitation effect in terms of sports ability recovery and rehabilitation [26,27,28].

Although VR and rehabilitation robot technology have shown broad prospects in modern rehabilitation medicine, their applications still face many challenges, such as high equipment costs, complex operations, and a large patient experience burden. In addition, most VR rehabilitation systems still need patients to wear more bulky physiological sensors, which will cause exercise restrictions for patients with inconvenience or recovery on the sick bed and increase the physical burden. Therefore, future research should focus on how to further optimize the experience of virtual reality technology and improve its accessibility and effectiveness in rehabilitation training.

The main contributions made in this study are as follows:

- The main contribution of this article is to propose a system based on virtual reality technology. By providing a more attractive and interactive rehabilitation training environment, the system significantly improves the participation and rehabilitation effect of patients. Compared with the traditional rehabilitation training method, the system can dynamically adjust the training plan according to the patient’s specific situation and help patients to correct the movement in time through real-time feedback mechanisms, thereby improving the personalization and accuracy of rehabilitation training.
- The research of this article is reflected in in-depth analysis and experimental verification of the application of virtual reality in the field of rehabilitation. Through experiments in patients with burns, this article not only confirms the effectiveness of the virtual reality rehabilitation training system but also shows its advantages in improving patient rehabilitation and improving the treatment experience. These findings provide an important empirical basis for the research and application of future virtual reality technology in the field of rehabilitation training.
- The work of this article in data collection and analysis is also worthy of attention. The system can collect a large amount of rehabilitation training data and use these data to provide detailed information and support for medical staff to help them optimize and personalize the treatment plan. This data-driven rehabilitation evaluation method not only improves the scientific and systematic nature of rehabilitation training but also provides new research ideas and tools for rehabilitation medicine.

## 3. System Architecture and Methods

### 3.1. System Framework Structure

**Figure 1:**
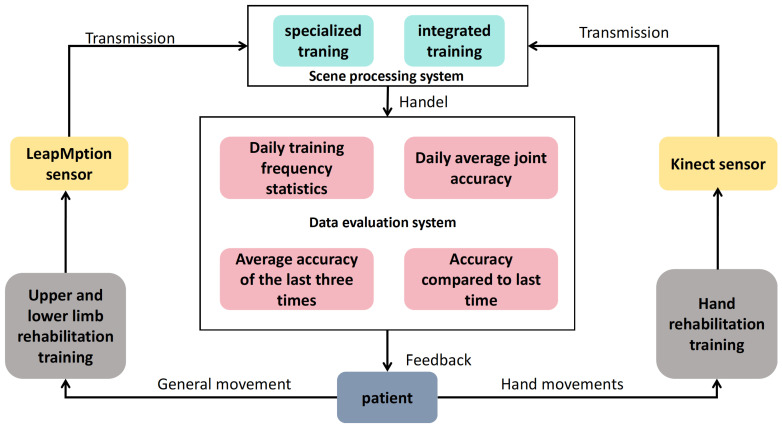
System framework structure diagram.

The system is rehabilitated for patients with dysfunction of upper and lower limbs. Patients make corresponding actions on the upper and lower limbs rehabilitation assessment and training equipment (hereinafter referred to as the rehabilitation evaluation equipment). For example, when the patient conducts rehabilitation training for the entire body’s large joints, the patient needs to face the equipment between 1 m and 3 m and perform rehabilitation training campaigns on the upper and lower limbs. The Kinect sensor device collects the patient’s movement trajectory and transmits it to Unity3D 2022.1 and 3DMAX 2021. In the scene processing system constructed by other simulated software, the scene processing system is mainly composed of two modules: special training and comprehensive training. Then, the scene processing system processes the rehabilitation training tasks and rehabilitation training requirements in the virtual scenario and transmits the processing results to the data evaluation system module. The data assessment system module can record the following according to the accuracy of the patient’s posture: the daily training number statistics, the daily average joint accuracy, the average accuracy of the past three times, and the accuracy compared with the last time. Finally, feedback on the processing results is provided to the patients. Similarly, when the patient conducts hand rehabilitation training, they only need to hang up at the front of the rehabilitation assessment equipment and perform the corresponding training exercise. The system frame structure is shown in Figure 1.

### 3.2. Hardware Equipment

**Figure 2:**
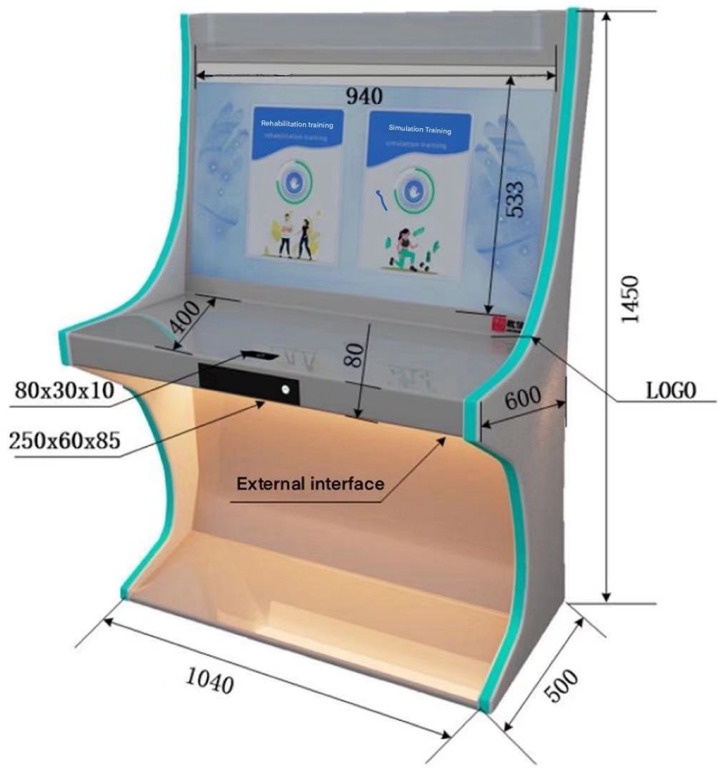
Upper, lower limb, and hand rehabilitation training assessment instrument.

The equipment used in this study was completely jointly developed by laboratory members. This product provides a highly comprehensive integrated treatment, training, and rehabilitation solution for patients with upper and lower limb movement disorders and hand dysfunction. The external structure of this product is made of metal to ensure product quality is strong, durable, stable, and safe. This product consists of a vertical all-in-one machine, a 43-inch large touch screen, and a touch monitor. It contains a Kinect2.0 somatosensory depth camera to detect and record patients doing motor dysfunction rehabilitation training and a Leap Motion gesture tracker to detect and record the medical treatment and rehabilitation status of patients with hand dysfunction. This product is independently developed throughout the entire process and is combined with software and hardware servers. Patient data can be transferred to the cloud and saved in real-time for subsequent doctor treatment needs. The full name of this equipment is the upper, lower limb, and hand Rehabilitation training assessment instrument, as shown in Figure 2. This product provides entertaining interactive training courses for patients with motor dysfunction. It uses a somatosensory camera to capture human movements. By comparing the accuracy of actual movements with standard movements, interaction with objects in the game training scene can be achieved, thereby improving the patient’s subjective initiative in rehabilitation training.

### 3.3. Hardware Equipment

The upper and lower limb gesture recognition sensor used is Kinect 2.0. This device is a depth-sensing camera launched by Microsoft  [29]. It uses a series of advanced technologies to realize its functions:

- Time-of-flight camera technology: Kinect 2.0 uses time-of-flight technology to measure the time it takes for light to be emitted from the camera to the surface of the object and back by sending infrared light pulses. This allows Kinect to accurately measure the depth of an object and create a depth image.
- High-resolution camera: Kinect 2.0 is equipped with a 1080p resolution color camera, capable of capturing clearer and more detailed images. This allows Kinect to more accurately recognize and track human movements and facial expressions.
- Multi-array microphone: Kinect 2.0 integrates multiple microphones to capture and locate spatial sounds. This enables the device to implement voice recognition and control functions.
- Bone-tracking technology: Kinect 2.0 uses advanced bone tracking algorithms to accurately capture the movement of human joints. This enables Kinect to track the user’s movements in real-time, enabling natural user interfaces and motion-sensing gaming.
- Infrared lighting and sensors: In order to work in low-light environments, Kinect 2.0 is equipped with infrared lighting and sensors that can work reliably under different lighting conditions.

**Figure 3:**
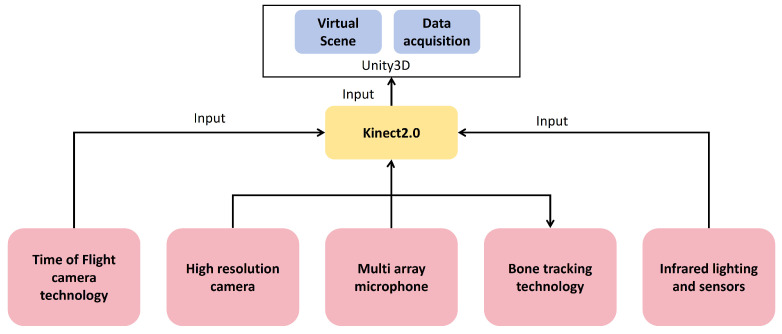
Upper, lower limb, and hand rehabilitation training assessment instrument.

The specific technology roadmap is shown in Figure 3.

The gesture recognition sensor used is Leap Motion, which uses a technology called “visual touchless gesture control”. The Leap Motion somatosensory interactor uses optical hand tracking technology and has two high-frame-rate grayscale infrared cameras with wide-angle lenses and four infrared LEDs. The optimal working environment is a lighting environment that can produce clear, high-contrast object outlines. The top layer The filter layer only allows infrared light waves to enter and exit and performs preliminary processing on the data collected by the camera to simplify the later calculation complexity; a binocular camera is used to extract the three-dimensional position of the hand through the principle of binocular stereo vision imaging and establish a three-dimensional model of the hand; using Grayscale cameras reduces the amount of computational data and increases the algorithm speed.

Based on the optimization of the above hardware and algorithms, Leap Motion can collect 200 frames of hand data per second with an accuracy of up to 0.01 mm. The trackable area of Leap Motion is in the shape of an inverted square pyramid in space, with a horizontal field of view of 140° and a vertical field of view of 120°. The interactive depth is between 10cm and 60cm, with a maximum of no more than 80 cm.

### 3.4. Collect Data and Interaction with Virtual Scenes

**Figure 4:**
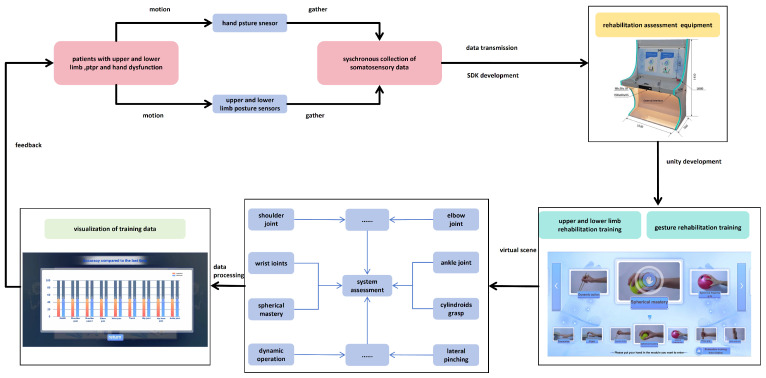
Interaction between the collected data and virtual scene.

Patients with upper and lower limb and hand dysfunction stand in front of the device and make corresponding movements. The patient’s motion data can be collected by the corresponding sensor and transmitted to the virtual program scene developed by Unity. The upper and lower limbs in this system provide are nine virtual scenes in the rehabilitation training, which are dedicated to training the following posture movements: neck, shoulder joint, shoulder joint 2, elbow joint, wrist joint, trunk, hip joint, hip–knee joint, and ankle joint. For the corresponding hand, there are 13 virtual scenes in the Ministry of Sports Rehabilitation training, which are dedicated to training the following posture movements: hanging, lifting, touching, pushing, hitting, dynamic operation, spherical grasp, spherical finger grip, columnar grasp, hook and pull, two-fingertip pinch, multiple-fingertip pinch, and side pinch. Patients undergo the above special training, and through system evaluation, visualized evaluation data are output and finally fed back to the patient so that the patient can adjust in real-time based on the training data to improve the accuracy of his rehabilitation training. Figure 4 shows the interaction between collected data and virtual scenes.

The interaction between the collected data and the virtual scene is shown in Figure 4.

### 3.5. Virtual Scene Design

**Figure 5:**
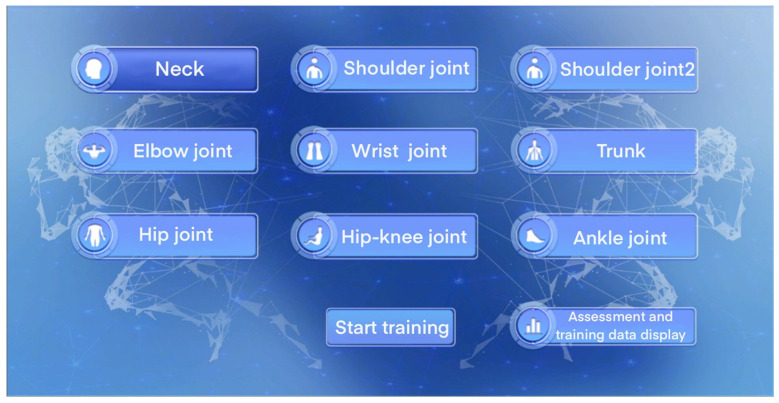
Trunk flexion exercises.

Interesting feedback can relieve patient fatigue during training and prevent treatments from becoming too tedious, which is critical for long-term recovery. This article designs a variety of simple and interesting biofeedback virtual environments to meet the needs of people with different degrees of respiratory impairment and provides users with training conditions for upper and lower limb rehabilitation and hand disorder rehabilitation. Through approachable and easy-to-operate training scenarios, user training operations are made convenient and concise. As shown in Figure 5, during the system development, this paper designed nine targeted training scenario selection interfaces for patients with upper and lower limb movement disorders.

**Figure 6:**
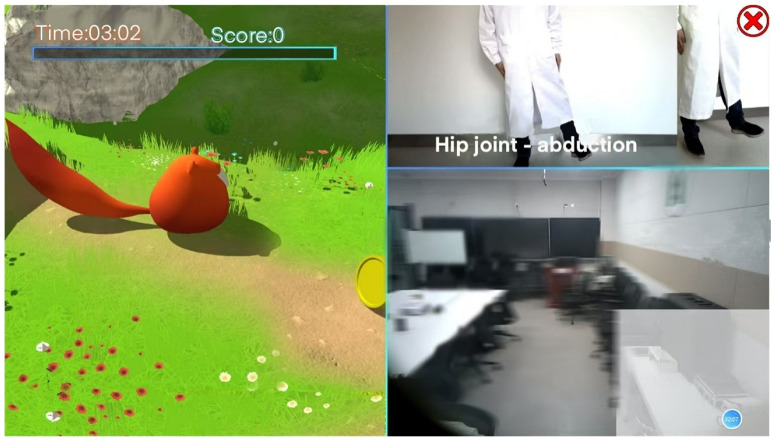
Interactive virtual rehabilitation training interface.

For example, in the trunk-flexion movement in special training, the patient stands in front of the device and makes the corresponding training posture according to the teaching video content prompted in the upper-right corner of the screen; in the lower-right corner, the patient can see his own movement trajectory information; on the left side of the screen is a virtual scene designed by this system. The small animals in the scene will give corresponding feedback according to the standard degree of the patient’s actions, such as jumping forward and picking up the gold coins in front. Prompts such as scores, time, and progress bars make the rehabilitation training more interesting for patients, as shown in Figure 6.

**Figure 7:**
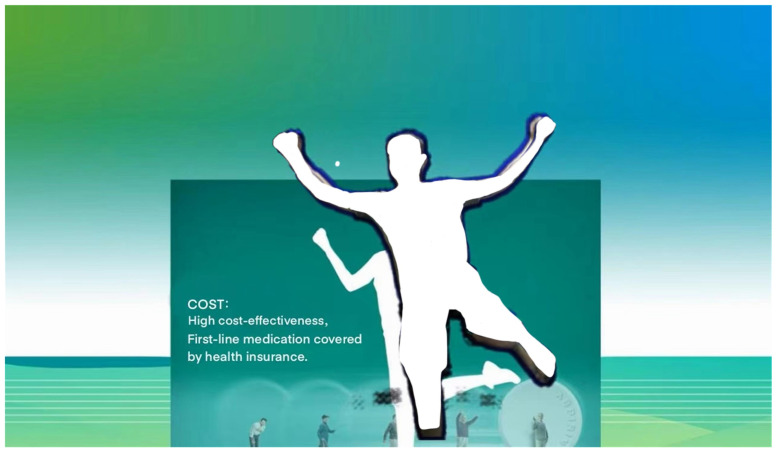
Fun training.

**Figure 8:**
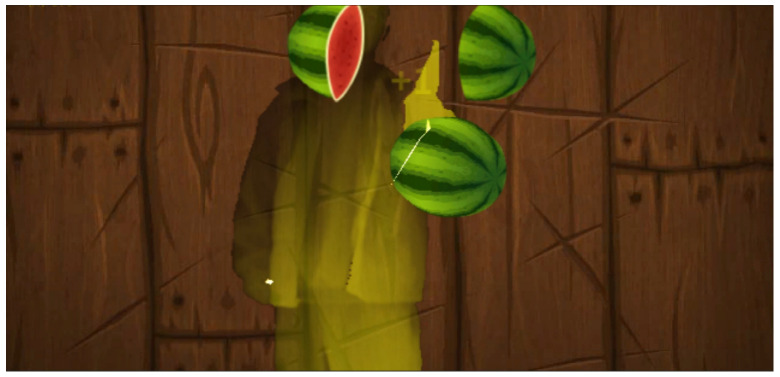
Cutting fruits based on movement.

Of course, in addition to nine different special trainings, this system also has some interesting random action exercises. Patients need to make corresponding actions according to the screen prompts. The distant screen will move from far to near as time goes by. If the patient’s actions are standard, there will be score feedback. If the patient’s actions are not standard, there will be a corresponding punishment mechanism, as shown in Figure 7. Similarly, the system has built-in a set of interesting training for cutting fruits based on the patient’s movements, as shown in Figure 8.

**Figure 9:**
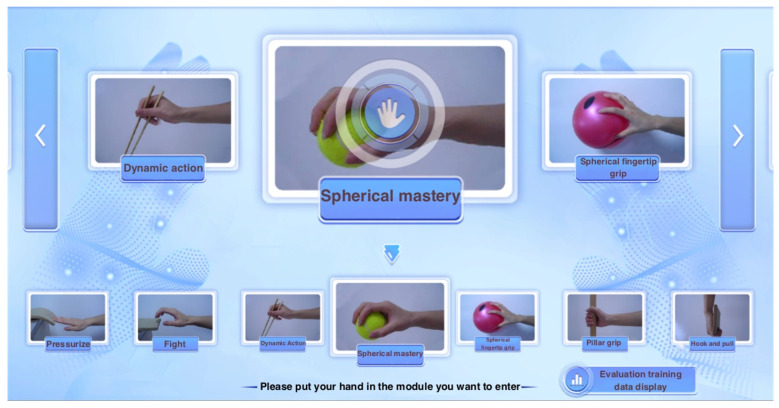
Thirteen types of special training.

Similarly, for patients with hand functional movement disorders, this article designed 13 targeted training scenarios, as shown in Figure 9.

**Figure 10:**
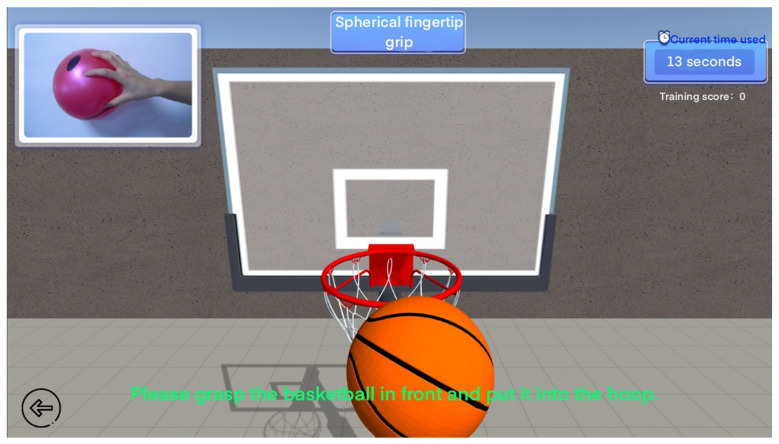
Ball fingertip grip special training.

**Figure 11:**
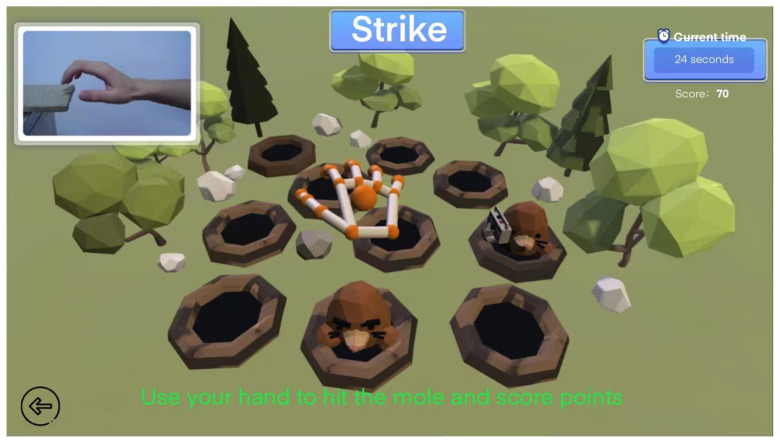
Special hitting training.

Here, we take the ball-shaped fingertip grip and hitting in the 13 special gesture training as examples. In the ball-shaped fingertip grip virtual scene, the patient will make grabbing movements according to the prompts in the scene and put the basketball into the ball. The more you put into the backboard within the specified time, the higher your score will be, as shown in Figure 10. In the virtual scene of special hitting training, patients need to perform standard hitting movements and hit the hamsters in the virtual scene with their hands. Within the specified time, the more hamsters hit, the higher the score, as shown in Figure 11. In this type of fun training, patients will not feel bored and will also record the training data in the game.

### 3.6. Training Data Collection and Visualization

#### Upper and Lower Limb Training Data Collection

Kinect can obtain the position coordinates of various joint points of the human body by processing depth data, such as the head, hands, feet, etc. Kinect can track up to two skeletons and can detect up to six people. The standing mode can track 20 joint points, and the sitting mode can track 10 joint points.

### 3.7. Hand Training Data Collection

When a hand enters the recognition area of Leap Motion, it will automatically track and output a series of data frames that are refreshed in real-time. Frame data are the core of Leap Motion. Each frame contains information about hand movements, such as all hands, fingers, pointables, tools, and gestures, and their position, velocity, direction, rotation angle, and other information. The hand model generated for each hand specifically includes the thumb, index finger, middle finger, ring finger, little finger, and wrist joint; for each finger, it includes the distal phalanx, middle phalanx, proximal phalanx, and metacarpal. Leap Motion simulates the skeletal joints of a real human hand and can achieve real-time, fast, and accurate hand tracking by updating information at each frame.

**Figure 12:**
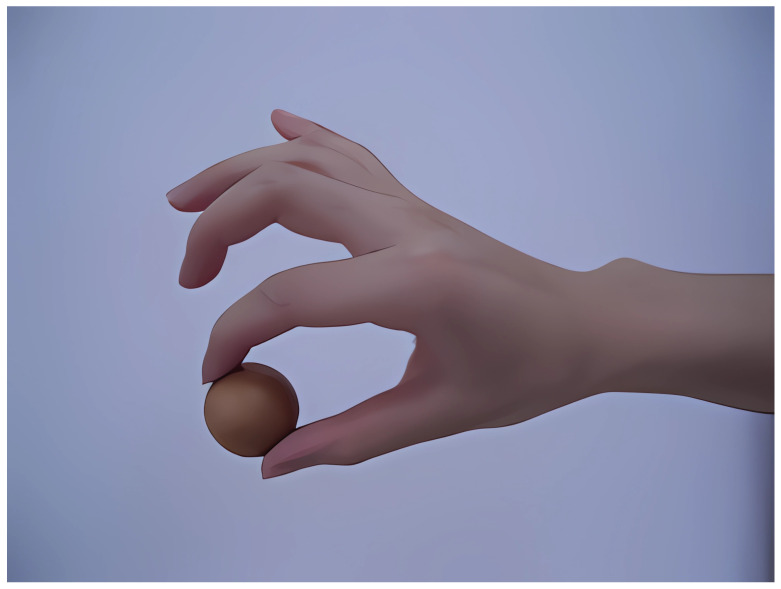
Standard movement of pinching with two fingertips.

Here, we take the special training in the system, two-fingertip pinching, as an example. We need to judge whether the patient makes the corresponding action and whether it is standard, as shown in Figure 12. We need to calculate and detect whether the positional characteristics of the third knuckle of the patient’s thumb are consistent with those of the third knuckle of each finger. The three-dimensional coordinate information of the third knuckle of the thumb is A(x_1_, y_1_, and z_1_), and the three-dimensional coordinate information of the third knuckle of the index finger is B (x_2_, y_2_, and z_2_).The distance d between two points A and B is the similarity between the thumb and the index finger. Based on the obtained d value, it can be judged whether the patient’s actions are standard.

(1) $$d=\sqrt{{\left(x_{1}-x_{2}\right)}^{2}+{\left(y_{1}-y_{2}\right)}^{2}+{\left(z_{1}-z_{2}\right)}^{2}}.$$

#### Visualized Data and Analysis

This system uses a variety of virtual reality gamification elements to embed rehabilitation training into virtual reality games and present patients’ performance through game scores, level progress, etc., thereby stimulating patients’ enthusiasm and participation. It also provides instant feedback, including sound prompts, visual feedback, and vibration feedback, are used to help patients adjust their movements and ensure correct posture and movement. Finally, the system generates rehabilitation training progress reports through the virtual reality system so that medical professionals can track the patient’s progress. These reports include graphics, charts, numerical data, etc.

**Figure 13:**
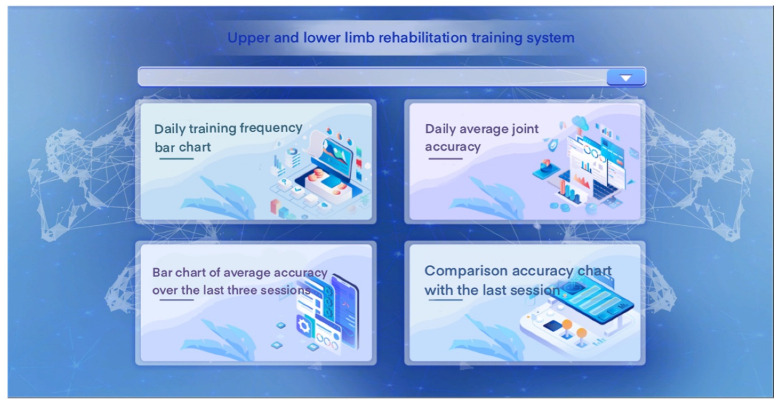
Rehabilitation training visualization data summary.

**Figure 14:**
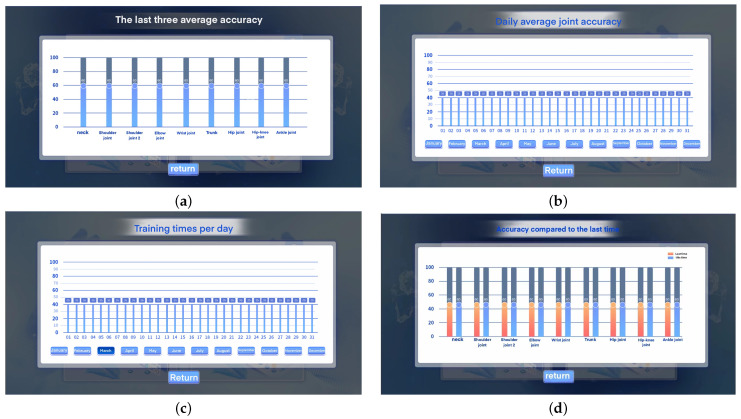
Patient rehabilitation training data record. Among them, Figure (**a**) is the interface for recording the average accuracy of the last three rehabilitation training sessions. Figure (**b**) is the interface for recording the average accuracy of joints in daily rehabilitation training. Figure (**c**) is the interface for recording the statistics of the number of daily rehabilitation training sessions. Figure (**d**) is the interface for comparing the accuracy with the last rehabilitation training session.

As shown in Figure 13, the system can record the patient’s rehabilitation training data, including a bar graph of the number of daily training sessions, a bar graph of the average joint accuracy of the past three sessions, and a chart comparing the accuracy with the last session. The detailed data display interface is shown in Figure 14. Among them, Figure 14a is an interface for recording the average accuracy of the last three rehabilitation training sessions. Figure 14b is an interface for recording the average joint accuracy of daily rehabilitation training. Figure 14c is an interface for recording the number of daily rehabilitation training sessions. Figure 14d is an interface for comparing the accuracy with the last rehabilitation training session.

When viewing the patient’s rehabilitation training data, the device then sends a data request to the cloud service. Here, the daily joint accuracy is taken as an example. See Algorithms 1 and 2.
**Algorithm 1** Uploading locally obtained data to the serverPost_Achievement*e*postUrl = postUrl_str + “/api/Hospital/uploadScore”string position = “position”string id = “id”string score = “score”WWWForm form = new WWWForm()form.AddField(position, “1”)form.AddField(id, “10000003”)form.AddField(score, *e*)UnityWebRequest request = UnityWebRequest.Post(postUrl, form)**yield** request.SendWebRequest()**if** request.isHttpError or request.isNetworkError **then**   Debug.LogError(request.error)**else**   string receiveContent = request.downloadHandler.text   Debug.Log(receiveContent)**end if**

**Algorithm 2** Obtaining the daily joint accuracy from the server
Post_Avg*e*postUrl = postUrl_str + “/api/Hospital/getJointAvgAccuracy”string position = “position”string id = “id”string month = “month”
WWWForm form = new WWWForm()form.AddField(position, “1”)form.AddField(id, “10000002”)form.AddField(month, *e*)
UnityWebRequest request = UnityWebRequest.Post(postUrl, form)**yield** request.SendWebRequest()**if** request.isHttpError or request.isNetworkError **then**   Debug.LogError(request.error)
**else**
   string receiveContent = request.downloadHandler.text   Debug.Log(receiveContent)
   string json = receiveContent   Data data = JsonUtility.FromJson<Data>(json)   Debug.Log(data.day1)
**end if**



**Figure 15:**
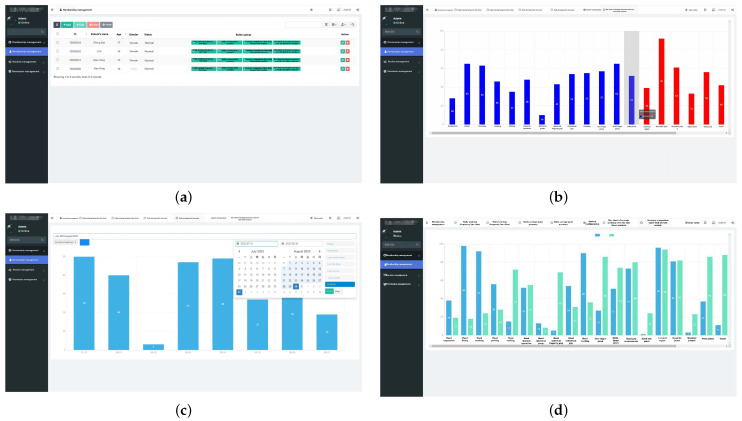
Detailed rehabilitation training data of patients.Among them, (**a**) is the background patient data management interface. (**b**) is the background patient rehabilitation data interface. (**c**) is the background patient rehabilitation detailed data interface. (**d**) is the background rehabilitation data comparison interface.

In addition to viewing the patient’s rehabilitation training data on the device, medical staff or patients can also view the patient’s detailed rehabilitation data by remotely accessing the server platform, as shown in Figure 15. Figure 15a shows the background patient data management interface. Figure 15b shows the background patient rehabilitation data. Figure 15c is the background patient rehabilitation detailed data interface. Figure 15d is the interface for comparison with the background rehabilitation data.

## 4. Experiments

### 4.1. Participants

In order to verify the effectiveness of the system, this study recruited 10 patients with medium burns to participate in the test. Before the test, we explained to all participants in detail the research purposes, processes, and possible risks and interests, and each participant signed a letter of informed consent and agreed to participate in this study. In order to ensure the safety of patients, we adopted a variety of measures, including gradual training, real-time monitoring, a user-friendly interface, comfort adjustment, security protocol, and data security protection to prevent discomfort. In the study, two professional staff were set up to guide the participants on how to correctly use the rehabilitation training system and conduct necessary training for the participants. Participants were divided into two groups: the first group was the control group, with five participants receiving routine traditional rehabilitation training, upper and lower limb rehabilitation training, and hand rehabilitation training. In the second observation group, the remaining five participants performed rehabilitation training in the virtual scene designed in this article. At present, burns are clinically divided into four levels: mild, moderate, severe, and extremely severe. Mild burns refer to a second-degree burn area of less than 10%, and there is no third-degree burn. Moderate burns refer to a second-degree burn area between 10% and 29% or a third-degree burn area of less than 10%. Severe burns refer to a second-degree burn area between 30% and 49%, or a third-degree burn area between 10% and 19%, or although the burn area is insufficient, it is accompanied by serious complications (such as shock, respiratory burns or other organ damage). Extremely severe burns refer to a second-degree burn area greater than 50%, or a third-degree burn area greater than 20%, or although it does not meet the above area standards, it is accompanied by serious complications [30].

To ensure the validity of the assessment, patients with other lung functions were excluded and participants met the following criteria:

- Meet the standards for moderate burns;
- Cooperate with training and other behaviors without resistance during rehabilitation training;
- Good compliance during rehabilitation training and tolerance during training.

Table 1 shows participant information. Participant screening was based on patients after moderate burns, mainly focusing on young and middle-aged people with an age distribution between 25 and 45 years old. In order to eliminate the interference of participants with other health conditions in the experiment, participants who had no other health conditions and were able to perform rehabilitation training on their own were selected for the experiment.

### 4.2. Experimental Process

After each breathing session, each participant received a questionnaire to fill out about their training experience. This scale examines participants’ feelings about training through the following questions [31]:

- Do you feel bored when training?
- During training, are you inattentive or distracted?
- During training, can you keep up with the training rhythm?

Each item in this experimental survey adopts a five-point Likert scale [32] with options ranging from 1 to 5; that is, strongly agree to strongly disagree. Each item is scored from 1 to 5, and the score is multiplied by the corresponding weight to obtain the final score, as shown in Table 2.

## 5. Results and Analysis

**Figure 16:**
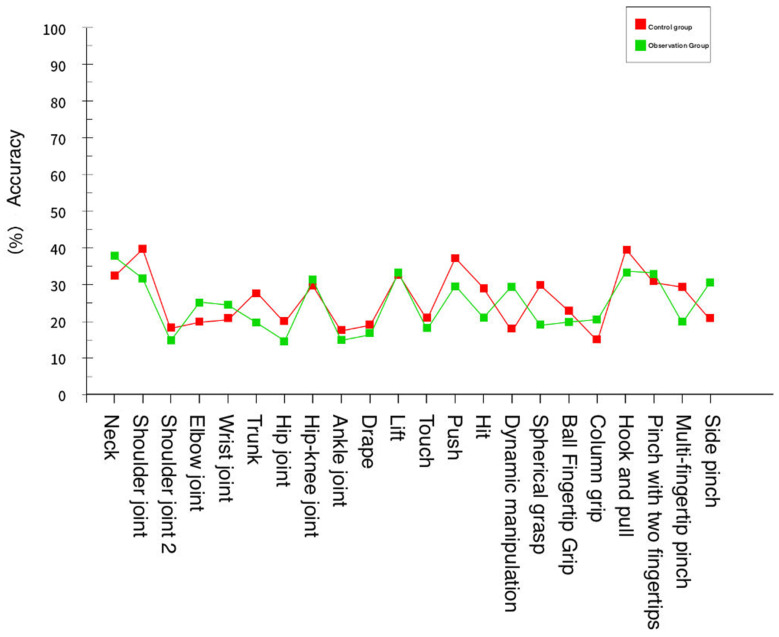
Movement standard of the control group and observation group before rehabilitation training.

During this rehabilitation training, the system recorded the changes in rehabilitation data parameters of each participant before and after training to measure the effectiveness of the training system. The experiment found that each participant’s rehabilitation training effect improved. Taking the data in Table 3 and Table 4 as an example, it can be seen that before traditional rehabilitation training or virtual reality rehabilitation training, the standard rates of rehabilitation training for members of the control group and observation group are similar, as shown in Figure 16.

The members of the control group performed one-on-one traditional rehabilitation training methods according to the rehabilitation trainer, while the members of the observation group used the virtual reality environment of this system for rehabilitation training and finally conducted a standardization test. The results are shown in Table 5 and Table 6.

**Figure 17:**
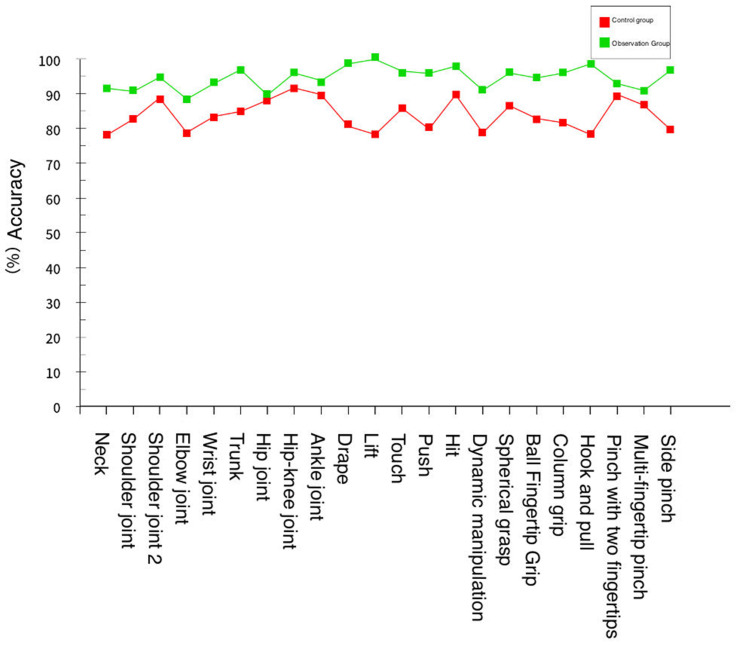
Movement standard of the control group and observation group after rehabilitation training.

It can be concluded from the experimental data provided by the training standard of the observation group members were higher overall than those of the control group members. The comparison of the rehabilitation training of both parties is shown in Figure 17.

**Figure 18:**
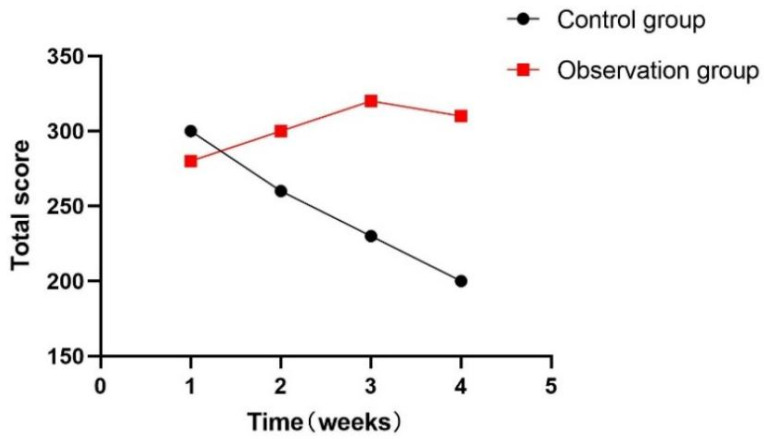
Total weekly boredom score.

**Figure 19:**
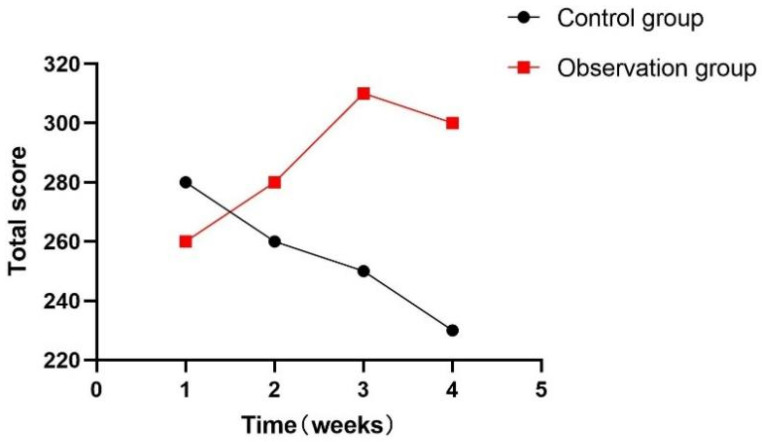
Total weekly inattention score.

**Figure 20:**
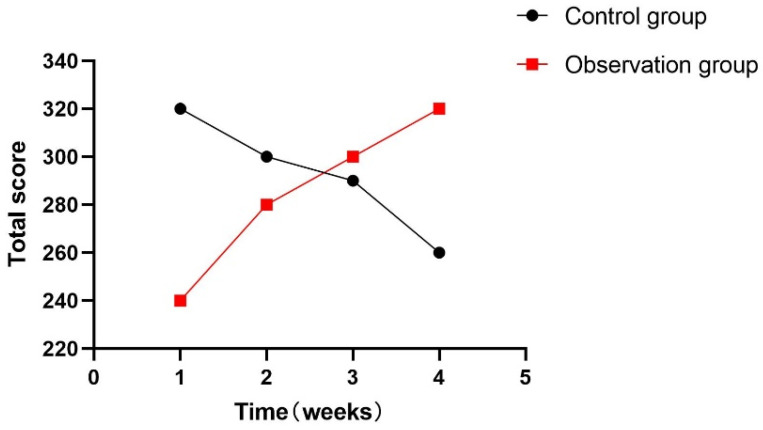
Total points for not keeping up each week.

At the same time, this experiment statistically summarized the feelings of each participant during the training process, as shown in Figure 18, Figure 19 and Figure 20. During the one-month experiment, subsequent rehabilitation training showed that compared with patients in the participation group, patients in the control group showed more problems, such as inattention, boredom, and an inability to keep up with the rhythm.

## 6. Conclusions

Traditional upper and lower limb or hand rehabilitation training requires a rehabilitation trainer to provide one-on-one guidance to the patient on rehabilitation movements. This training is expensive and inconvenient for the patient. In contrast, this article provides an evaluation system for upper and lower limb and hand rehabilitation training based on virtual reality technology, which embodies the method of combining virtual reality technology with rehabilitation training and also realizes the visualization and traceability of rehabilitation training data, providing real-time feedback to help patients better understand and adjust their movements. This timely feedback helps to improve the quality of rehabilitation training.

The purpose of this research system is to improve the rehabilitation training quality of patients with motor disorders of upper and lower limb and hand dysfunction and realize a more convenient, practical, and interesting self-chemical training method. Through this experimental equipment and Kinect and LeapMotion data sensors, we have realized the research on methods of combining human upper and lower limb movements and hand movements with virtual scenes and interacting in real-time. We conduct data analysis on the data collected after human movement, use human movement data to drive changes in the virtual scene, and realize the interaction between human movement data and virtual scenes. We use data visualization algorithms to quantify and visualize movement data to evaluate the effectiveness of patient rehabilitation training. In the virtual scene of rehabilitation training, interesting methods such as tasks, scores, and plots are used to guide patients to continue rehabilitation training in a comfortable process, thereby completing specific rehabilitation training tasks and goals. The system is evaluated from two aspects: training effect and user experience. Comparing the experimental results and experiences of 10 participants, the results show that compared with the traditional one-to-one guidance of rehabilitation trainers on patients in rehabilitation movements, this research system has better training effects and experiences, and rehabilitation training is more active.

The experimental results show that virtual reality technology can significantly improve the rehabilitation effect in upper and lower limb and hand rehabilitation training. Patients’ rehabilitation training in the virtual simulation environment shows greater participation and enthusiasm. Compared with traditional methods, the rehabilitation effect is more significant. Virtual reality technology injects more entertainment and interactivity into rehabilitation training, thereby significantly enhancing patients’ motivation. Patients are more willing to invest in the virtual simulation environment, making the recovery process more pleasant, and improving treatment compliance. The experimental results support the effectiveness of personalized rehabilitation programs based on virtual reality technology. The system can dynamically adjust rehabilitation training according to the patient’s specific needs and progress, making the treatment closer to the patient’s individual differences and improving the pertinence and effectiveness of the treatment. The real-time feedback provided by the virtual simulation system is crucial to the patient’s rehabilitation process. Through real-time movement monitoring and feedback, this system makes it easier for patients to correct improper movements and improve motor skills, thus accelerating the rehabilitation process.

According to the experimental results, the upper limbs and lower extremity rehabilitation training evaluation system based on virtual reality technology has shown great advantages in many ways, introducing innovative treatment methods into the field of rehabilitation. However, further long-term studies and large-scale applications still need to be conducted to more fully evaluate the long-term effects of the system and long-term patient acceptance.

## 7. Limitation and Challenges

Although virtual reality technology has shown great potential in rehabilitation training, its high cost and technical complexity may indeed hinder the widespread application of this technology, especially in resource-limited environments.

Doctors suggest that future research should focus more on individual differences and develop personalized adaptive training programs to improve patient acceptance and compliance. Additionally, doctors emphasize the importance of multidisciplinary collaboration, including cooperation among physicians, physical therapists, engineers, and patients, to ensure that the system’s development meets actual clinical needs and the personal preferences of patients. They also recommend conducting long-term follow-up studies to evaluate the system’s impact on patients’ long-term rehabilitation and whether it can continuously improve patients’ functional status.

In response to the challenges posed by high costs, technical complexity, and patient adaptability in virtual reality technology for rehabilitation training, we are committed to reducing costs and simplifying technical operations to enhance the system’s accessibility. Based on doctors’ recommendations, we will develop personalized rehabilitation plans to meet the specific needs of different patients, thereby improving their acceptance and compliance. We will also pay close attention to data security and privacy protection issues, ensuring that effective measures are taken to safeguard patients’ personal information. With the continuous advancement of technology, we will continue to conduct research to assess the long-term effects of virtual reality technology in the field of rehabilitation, address existing shortcomings, and ensure that our system can provide long-term and effective rehabilitation support for patients.

We believe that by addressing these limitations and undertaking future work, the virtual reality-based upper and lower limb training assessment system will bring greater value to the field of rehabilitation medicine and provide higher-quality rehabilitation services for patients.

## Figures and Tables

**Table 1:** Participants’ information.

Participant ID	Gender	Age	Other Medical History	Burn Degree	Ability to Train Independently
1	Male	25	No	Moderate	Yes
2	Male	40	No	Moderate	Yes
3	Male	34	No	Moderate	Yes
4	Male	38	No	Moderate	Yes
5	Male	29	No	Moderate	Yes
6	Female	31	No	Moderate	Yes
7	Female	40	No	Moderate	Yes
8	Female	42	No	Moderate	Yes
9	Female	26	No	Moderate	Yes
10	Female	30	No	Moderate	Yes

**Table 2:** Experimental training feeling scale.

Serial Number	Question	Very Much Agree	Agree	General	Disagree	Strongly Opposed
1	Do you feel bored when training?	1	2	3	4	5
2	Are you distracted and unable to concentrate during training?	1	2	3	4	5
3	When training, are you unable to keep up with the training pace?	1	2	3	4	5

**Table 3:** Accuracy rate of members in the control group before rehabilitation training.

Rehabilitation Movements	Total Number of Movements	Average Number of Exercises per Person	Total Number of Criteria Reached	Standard Rate
Neck (upper and lower limbs)	100 times	20 times	32	32%
Shoulder joint (upper and lower limbs)	100 times	20 times	40	40%
Shoulder joint 2 (upper and lower limbs)	100 times	20 times	18	18%
Elbow joint (upper and lower limbs)	100 times	20 times	20	20%
Wrist joint (upper and lower limbs)	100 times	20 times	21	21%
Trunk (upper and lower limbs)	100 times	20 times	28	28%
Hip joint (upper and lower limbs)	100 times	20 times	20	20%
Hip-knee joint (upper and lower limbs)	100 times	20 times	30	30%
Ankle joint (upper and lower limbs)	100 times	20 times	17	17%
Drape (hand)	100 times	20 times	19	19%
Lift				
(hand)	100 times	20 times	33	33%
touch				
(hand)	100 times	20 times	21	21%
Push				
(hand)	100 times	20 times	37	37%
Hit				
(hand)	100 times	20 times	29	29%
Dynamic manipulation (hands)	100 times	20 times	18	18%
Spherical grasp				
(hand)	100 times	20 times	30	30%
Ball Fingertip Grip (Hand)	100 times	20 times	24	24%
Column grip				
(hand)	100 times	20 times	15	15%
Hook and pull				
(hand)	100 times	20 times	40	40%
Pinch with two fingertips (hand)	100 times	20 times	31	31%
Multi-fingertip pinch (hand)	100 times	20 times	29	29%
Side pinch (hand)	100 times	20 times	21	21%

**Table 4:** Accuracy rate of members of the observation group before rehabilitation training.

Rehabilitation Movements	Total Number of Movements	Average Number of Exercises per Person	Total Number of Criteria Reached	Standard Rate
Neck (upper and lower limbs)	100 times	20 times	38	38%
Shoulder joint (upper and lower limbs)	100 times	20 times	32	32%
Shoulder joint 2 (upper and lower limbs)	100 times	20 times	15	15%
Elbow joint (upper and lower limbs)	100 times	20 times	25	25%
Wrist joint (upper and lower limbs)	100 times	20 times	24	24%
Trunk (upper and lower limbs)	100 times	20 times	20	20%
Hip joint (upper and lower limbs)	100 times	20 times	15	15%
Hip-knee joint (upper and lower limbs)	100 times	20 times	32	32%
Ankle joint (upper and lower limbs)	100 times	20 times	15	15%
Drape				
(hand)	100 times	20 times	17	17%
Lift				
(hand)	100 times	20 times	34	34%
touch				
(hand)	100 times	20 times	22	22%
Push				
(hand)	100 times	20 times	30	30%
Hit				
(hand)	100 times	20 times	21	21%
Dynamic manipulation (hands)	100 times	20 times	30	30%
Spherical grasp				
(hand)	100 times	20 times	19	19%
Ball Fingertip Grip (Hand)	100 times	20 times	20	20%
Column grip				
(hand)	100 times	20 times	21	21%
Hook and pull				
(hand)	100 times	20 times	34	34%
Pinch with two fingertips (hand)	100 times	20 times	33	33%
Multi-fingertip pinch (hand)	100 times	20 times	20	20%
Side pinch (hand)	100 times	20 times	31	31%

**Table 5:** Correct rate of members of the control group after rehabilitation training.

Rehabilitation Movements	Total Number of Movements	Average Number of Exercises per Person	Total Number of Criteria Reached	Standard Rate
Neck (upper and lower limbs)	100 times	20 times	78	78%
Shoulder joint (upper and lower limbs)	100 times	20 times	83	83%
Shoulder joint 2 (upper and lower limbs)	100 times	20 times	89	89%
Elbow joint (upper and lower limbs)	100 times	20 times	79	79%
Wrist joint (upper and lower limbs)	100 times	20 times	83	83%
Trunk (upper and lower limbs)	100 times	20 times	85	85%
Hip joint (upper and lower limbs)	100 times	20 times	88	88%
Hip-knee joint (upper and lower limbs)	100 times	20 times	92	92%
Ankle joint (upper and lower limbs)	100 times	20 times	90	90%
Drape				
(hand)	100 times	20 times	81	81%
Lift				
(hand)	100 times	20 times	78	78%
touch				
(hand)	100 times	20 times	86	86%
Push				
(hand)	100 times	20 times	80	80%
Hit				
(hand)	100 times	20 times	90	90%
Dynamic manipulation (hands)	100 times	20 times	79	79%
Spherical grasp				
(hand)	100 times	20 times	87	87%
Ball Fingertip Grip (Hand)	100 times	20 times	83	83%
Column grip				
(hand)	100 times	20 times	82	82%
Hook and pull				
(hand)	100 times	20 times	78	78%
Pinch with two fingertips (hand)	100 times	20 times	90	90%
Multi-fingertip pinch (hand)	100 times	20 times	87	87%
Side pinch (hand)	100 times	20 times	80	80%

**Table 6:** The accuracy rate of members of the observation group after rehabilitation training.

Rehabilitation Movements	Total Number of Movements	Average Number of Exercises per Person	Total Number of Criteria Reached	Standard Rate
Neck (upper and lower limbs)	100 times	20 times	92	92%
Shoulder joint (upper and lower limbs)	100 times	20 times	91	91%
Shoulder joint 2 (upper and lower limbs)	100 times	20 times	95	95%
Elbow joint (upper and lower limbs)	100 times	20 times	89	89%
Wrist joint (upper and lower limbs)	100 times	20 times	93	93%
Trunk (upper and lower limbs)	100 times	20 times	97	97%
Hip joint (upper and lower limbs)	100 times	20 times	90	90%
Hip-knee joint (upper and lower limbs)	100 times	20 times	96	96%
Ankle joint (upper and lower limbs)	100 times	20 times	94	94%
Drape				
(hand)	100 times	20 times	99	99%
Lift				
(hand)	100 times	20 times	100	100%
touch				
(hand)	100 times	20 times	97	97%
Push				
(hand)	100 times	20 times	96	96%
Hit				
(hand)	100 times	20 times	98	98%
Dynamic manipulation (hands)	100 times	20 times	91	91%
Spherical grasp				
(hand)	100 times	20 times	96	96%
Ball Fingertip Grip (Hand)	100 times	20 times	95	95%
Column grip				
(hand)	100 times	20 times	96	96%
Hook and pull				
(hand)	100 times	20 times	99	99%
Pinch with two fingertips (hand)	100 times	20 times	93	93%
Multi-fingertip pinch (hand)	100 times	20 times	91	91%
Side pinch (hand)	100 times	20 times	97	97%

## Data Availability

Data are contained within the article.
